# *S-LOCUS EARLY FLOWERING 3* Is Exclusively Present in the Genomes of Short-Styled Buckwheat Plants that Exhibit Heteromorphic Self-Incompatibility

**DOI:** 10.1371/journal.pone.0031264

**Published:** 2012-02-01

**Authors:** Yasuo Yasui, Masashi Mori, Jotaro Aii, Tomoko Abe, Daiki Matsumoto, Shingo Sato, Yoriko Hayashi, Ohmi Ohnishi, Tatsuya Ota

**Affiliations:** 1 Graduate School of Agriculture, Kyoto University, Sakyou-ku, Kyoto, Japan; 2 Research Institute for Bioresources and Biotechnology, Ishikawa Prefectural University, Nonoichi-machi, Ishikawa, Japan; 3 Faculty of Applied Life Science, Niigata University of Pharmacy and Applied Life Science, Akiha-ku, Niigata, Japan; 4 Nishina Center for Accelerator-Based Science, RIKEN, Wako, Saitama, Japan; 5 Department of Evolutionary Studies of Biosystems, The Graduate University for Advanced Studies, Hayama, Kanagawa, Japan; University of Melbourne, Australia

## Abstract

The different forms of flowers in a species have attracted the attention of many evolutionary biologists, including Charles Darwin. In *Fagopyrum esculentum* (common buckwheat), the occurrence of dimorphic flowers, namely short-styled and long-styled flowers, is associated with a type of self-incompatibility (SI) called heteromorphic SI. The floral morphology and intra-morph incompatibility are both determined by a single genetic locus named the *S*-locus. Plants with short-styled flowers are heterozygous (*S/s*) and plants with long-styled flowers are homozygous recessive (*s/s*) at the *S*-locus. Despite recent progress in our understanding of the molecular basis of flower development and plant SI systems, the molecular mechanisms underlying heteromorphic SI remain unresolved. By examining differentially expressed genes from the styles of the two floral morphs, we identified a gene that is expressed only in short-styled plants. The novel gene identified was completely linked to the *S*-locus in a linkage analysis of 1,373 plants and had homology to *EARLY FLOWERING 3*. We named this gene *S-LOCUS EARLY FLOWERING 3* (*S*-*ELF3*). In an ion-beam-induced mutant that harbored a deletion in the genomic region spanning *S-ELF3*, a phenotype shift from short-styled flowers to long-styled flowers was observed. Furthermore, *S-ELF3* was present in the genome of short-styled plants and absent from that of long-styled plants both in world-wide landraces of buckwheat and in two distantly related *Fagopyrum* species that exhibit heteromorphic SI. Moreover, independent disruptions of *S-ELF3* were detected in a recently emerged self-compatible *Fagopyrum* species and a self-compatible line of buckwheat. The nonessential role of *S-ELF3* in the survival of individuals and the prolonged evolutionary presence only in the genomes of short-styled plants exhibiting heteromorphic SI suggests that *S-ELF3* is a suitable candidate gene for the control of the short-styled phenotype of buckwheat plants.

## Introduction

**Figure 1:**
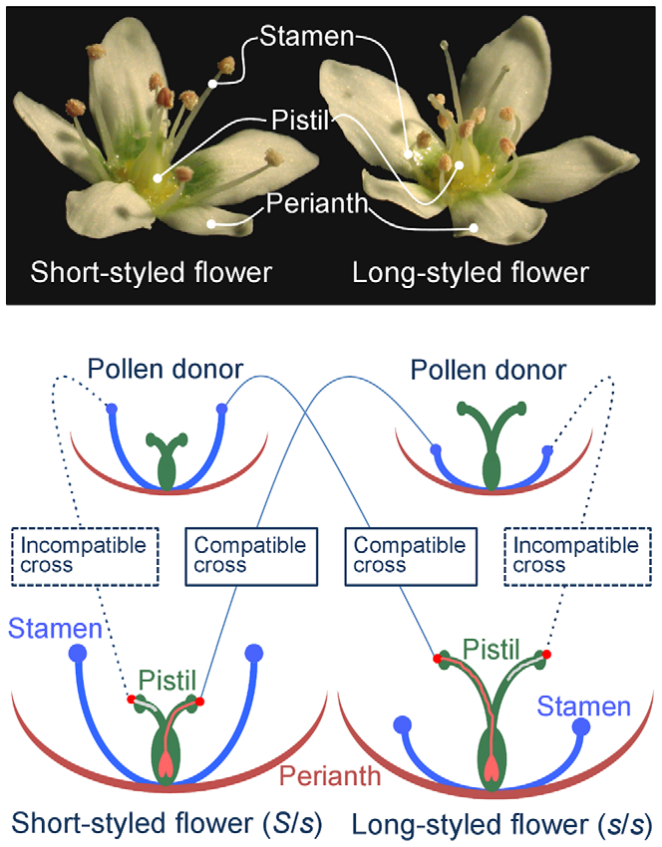
Dimorphic flowers of buckwheat and schematic presentation of the intra-morph incompatibility response in buckwheat. Short-styled flowers of buckwheat have long stamens and vice versa. A pollen grain from a long-styled plant germinates and the pollen tube successfully elongates to reach the ovary in the pistil of a short-styled plant, whereas it germinates but fails to elongate in the style of long-styled flower.

Heteromorphic or heterostylous self-incompatibility (SI), which has been observed in 28 angiosperm families, is associated with distinct variations in floral features, such as style length, stamen length, pollen size and intramorph incompatibility [1], [2]. *Fagopyrum esculentum* (common buckwheat) is an agronomically important species that exhibits heteromorphic SI (Fig. 1). The floral morphology and the intra-morph incompatibility response in *F. esculentum* is determined by a single genetic locus named the *S*-locus, where plants with short- and long-styled flowers are heterozygotes of the *S* and *s* haplotypes and homozygotes of the *s* haplotype, respectively [3], [4].

The molecular mechanism underlying plant SI has been investigated over the past few decades. Three homomorphic SI systems, in which no flower morph variations accompany SI, have been examined in detail. These include sporophytic SI, which is based on the SP11/SCR-SRK-mediated signaling cascade, in the mustard family [5]–[9]; gametophytic SI, which is based on the SLF-SFB/S-RNase system, in the potato, rose, and plantain families [10]–[12]; and gametophytic SI, which is based on the PrpS/PrsS system, in the poppy family [13], [14]. In heteromorphic SI of buckwheat, the genotype of sporophytes determines the incompatibility type; i.e., plants that have the *S/s* genotype produce only pollen that exhibit the *S* phenotype and not the *s* phenotype. Previous studies pointed out that heteromorphic SI is unlikely to be related to homomorphic sporophytic SI based on the phylogenetic independence between these two types of SI, and on the difference in timing of pollen rejection between these two systems [15]–[17]. Therefore, the molecular mechanism underlying SI in buckwheat is expected to be novel.

Recent molecular biological and genetic studies in the heterostylous *Primula* [18]–[21] and *Turnera* [22]–[24] species have advanced our understanding of the molecular mechanisms of heteromorphic SI. In *Primula*, in which at least three genes are present at the *S*-locus [4], [25], molecular markers linked to the *S*-locus, including two genes differentially expressed between the two morphs, were identified [18], [19]. Furthermore, BAC contigs for *S*-linked markers were successfully assembled using *P. vulgaris* bacterial artificial chromosome (BAC) genomic libraries [20], [21]. A large-scale expressed sequence tag (EST) analysis of the two floral morphs is also underway [21]. In *Turnera*, a high-resolution linkage map [22] and a deletion map [23] of the region spanning the *S*-locus were constructed. Positional cloning using the *S*-linked markers furthermore identified BACs containing the genomic region of the *s* haplotype [24]. Despite this recent progress, no genes responsible for heteromorphic SI have been identified in either species and the molecular mechanism controlling heteromorphic SI remains unknown [21], [24], [26].

Regarding buckwheat heteromorphic SI, random amplified polymorphic DNA (RAPD) markers and amplified fragment length polymorphism (AFLP) markers were identified around the *S*-locus [27], [28]. Two-dimensional electrophoresis also detected several proteins that were specifically expressed in long or short pistils [29]. An interspecific cross between buckwheat and a self-compatible wild species, *F. homotropicum*, which has an *S^h^* allele at the *S*-locus, generated self-compatible lines (KSC2 and Kyushu PL4). An analysis of the floral morphs and self-incompatibility responses of KSC2 suggested the presence of multiple genes at the *S*-locus [30]. Recently, we constructed a BAC genomic library of 7.6X coverage and initiated a search for genes that control heteromorphic SI [31]. One eminent advantage of studying buckwheat is the availability of numerous landraces that have been cultivated in the temperate zones of the northern hemisphere and the presence of heteromorphic SI and homomorphic self-compatible (SC) species in the same genus. It is generally difficult to conduct a potent association analysis in crop plants, due to their complex population structure [32]. Since an analysis of protein variation among worldwide populations revealed that there is no significant local differentiation in buckwheat [33], an association study using landraces was undertaken to supplement a linkage analysis to identify the genes responsible for heteromorphic SI. In addition, exhaustive phylogenetic studies revealed that at least ten species of two anciently diverged groups of *Fagopyrum* display heteromorphic SI and only a few species display homomorphic SC [34]–[37]. Heteromorphic SI has persisted since the emergence of the genus, but independent recurrent mutations have resulted in the occasional acquisition of self-compatibility and loss of heteromorphy. Since no established method is available for transforming buckwheat, evolutionary analysis using these plants is an instructive alternative for examining the functional importance of genes in the heteromorphic SI system of buckwheat.

Here, we sought to determine the molecular basis of buckwheat heteromorphic SI by identifying the primary factor(s) involved in this process. By integrating a variety of genetic and molecular approaches, including transcriptome analysis, mutagenesis screening and evolutionary genetic analysis, we identified one candidate gene, *S-LOCUS EARLY FLOWERING 3* (*S-ELF3*).

## Results

### Transcriptome analysis of stylar RNAs

**Figure 2:**
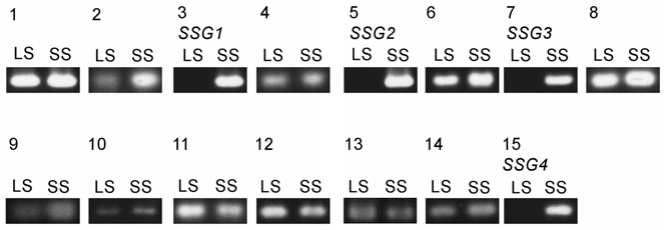
Expression of genes selected by in *silico* subtraction as determined by RT-PCR. The expression of genes corresponding to the 15 contigs selected by *in silico* subtraction was examined by RT-PCR using cDNA from long styles (LS) and short styles (SS) as templates. See Table S1 for RT-PCR primers.

To identify a *S*-haplotype-specific and/or short-style-specific gene (SSG) by subtracting genes expressed in long-styled plants (*s*/*s* genotype) from genes expressed in short-styled plants (*S*/*s* genotype), RNA was isolated from each of the two distinct floral types of a sib-mating line of buckwheat. The plant line utilized was derived from a single pair of short-styled and long-styled plants and sib-mating was conducted for generations (BC_1_-F_5_); therefore, the genetic difference between plants of different morphs was largely reduced, which facilitated the screening for differentially expressed genes at the *S*-locus. Furthermore, this line was previously used to construct a BAC genomic library [33], and any SSG molecular marker identified could readily be used for subsequent chromosome walking, because the library contained the *S*-locus genomic region of the *S* haplotype derived from a single chromosome. Total RNA isolated from the two distinct types of floral styles was then separately subjected to high-throughput sequence analysis using an Illumina GAII sequencer. The analysis yielded 7,371,322 pairs of 50mer reads for short-styled plants, from which 41,599 contigs of various length (61 bp–5,334 bp) were assembled by the Velvet program. Analysis of RNA isolated from long-styled plants yielded 2,522,996 pairs of 50mer reads and 3,938,668 pairs of 51mer reads, which were used to examine if the fragments assembled for short-styled plants contained the reads obtained from long-styled plants. This *in silico* subtraction procedure eliminated most of contigs and only 15 contigs remained as SSG candidates. RT-PCR analysis of these 15 contigs showed that only four were exclusively expressed in short styles. We tentatively named these four genes *SSG1*–*SSG4* (Table S1, Fig. 2).

### Analysis of a chimeric plant generated by ion-beam mutagenesis

**Figure 3:**
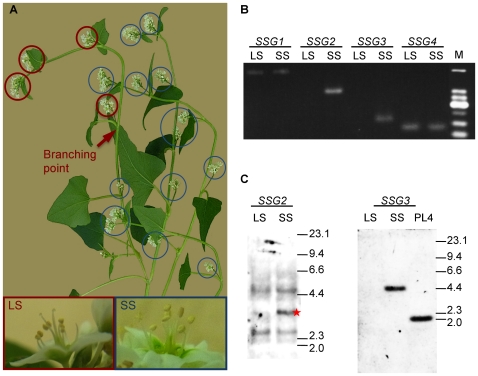
Analysis of a chimeric mutant plant and a short-styled-specific gene (SSG). (A) A chimeric plant generated by ion-beam mutagenesis. Red and blue circles indicate long-styled (LS) and short-styled (SS) flowers, respectively. (B) PCR amplification of gene fragments for *SSG1-SSG4* using genomic DNA isolated from the chimeric plant as template. M, molecular marker (25-bp DNA ladder, Invitrogen). (C) Southern blot analysis of *SSG2* and *SSG3* (*S-ELF3*) using genomic DNA isolated from the Kitawase cultivar (LS, SS) or Kyushu PL4 (PL4) as template. The star indicates the band corresponding to *SSG2*. Fragment sizes of the λ*Hin*dIII marker shown at the right are in kb.

During the mutagenesis screening of buckwheat, a single chimeric plant possessing a branch that sets long-styled flowers on a short-styled plant was obtained from among 1,400 M_1_ plants (Fig. 3A). In spite of our great interest in its SI phenotype, we were unable to determine what kind of SI response the long-styled flowers of this plant showed, because mating using pollen grains from these flowers was unsuccessful on the pistils of both long- and short-styled plants, and mating using pollen grains from both long- and short-styled plants was unsuccessful on the pistils of these flowers. Nevertheless, PCR analysis of the genomic DNA of this chimeric plant yielded intriguing results. PCR successfully amplified *SSG2* and *SSG3* from the total DNA of the short-styled part of the plant but not from the total DNA of the long-styled part of the plant (Fig. 3B). On the other hand, PCR successfully amplified *SSG1* and *SSG4* from DNA isolated from both short- and long-styled parts of the plant. Considering that *SSG2* and *SSG3* are tightly linked and separated by about 100 kb (see below), this result suggests that somatic deletion of the region that includes *SSG2* and *SSG3* occurred in the chimeric plant, and raises the possibility that these genes are located at the *S*-locus, which determines the floral phenotype. Interestingly, the presence of *SSG3* in the plant genome with short- but not long-styled flowers was not limited to the chimeric mutant, but was also observed in a normal plant of the Kitawase cultivar, as illustrated by Southern blot analysis (Fig. 3C). On the other hand, multiple bands were present in the Southern blot analysis of *SSG2* using DNA isolated from both short- and long-styled plants, demonstrating that the genome of long-styled plants contained at least one gene closely related to *SSG2*.

### Characterization of *SSG3*

RACE and RT-PCR analysis of *SSG3* identified cDNAs that encode a homolog of *Arabidopsis thaliana* ELF3 (*Ath*ELF3), which consists of 661 amino acids (Fig. S1A). Whereas the deduced amino acid sequence does not contain a signal peptide, it does contain a predicted monopartite nuclear localization signal, RVPLRKKKKAL, in the middle, indicating that, like AthELF3, it functions in the nuclear compartment. It should also be noted that two conserved peptide motifs of ELF3, i.e., GGP(R/K)(P/A)PPRNKMA, near the N terminus, and (A/V)(M/A/V)(K/R)IF(R/Q)SIQXER, near the C terminus, were present. The transcriptome analysis of stylar RNA revealed another homolog of *Ath*ELF3 (Fig. S1B, ELF3). Phylogenetic analysis based on the deduced amino acid sequences (Fig. S1C) showed that the latter homolog is evolutionarily closer to *Ath*ELF3 and that *SSG3* is not an ortholog but a paralog of *ELF3*.

**Figure 4:**
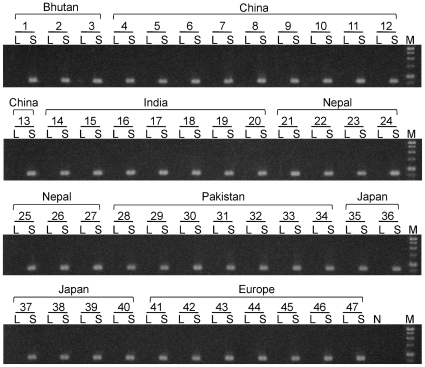
PCR survey of *S-ELF3* (*SSG3*) in 47 buckwheat landraces and modern cultivars. The numbering of individual plants corresponds to that shown in Table S2. L, long-styled plant. S, short-styled plant. N, negative control. M, 1-kb DNA ladder (GenDireX).

**Figure 5:**
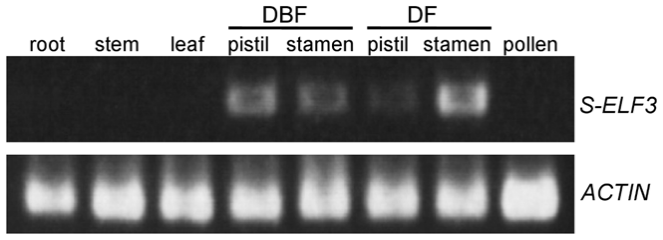
Expression analysis of *S-ELF3* transcripts. cDNA prepared from roots, stems, leaves, pistils, stamens and pollen was used for RT-PCR analysis. *ACTIN* was amplified as a loading control. DBF: one day before flowering. DF: the day of flowering.

In the linkage analysis, floral morphology was used to examine the linkage relationship of the *S*-locus to *S-ELF3*. No recombination between *SSG3* and the short-styled phenotype was detected in 1,373 plants of the sib-mating line, suggesting that there is a small genetic distance, if any, between this gene and the *S*-locus (0.0–0.4 cM). PCR analysis of 47 short-styled and long-styled pairs of buckwheat landraces and modern cultivars collected from Asia to Europe showed that there is a complete association between the presence of this gene and the type of flower exhibited; all plants with short-styled flowers but none with long-styled flowers possessed *SSG3* (Fig. 4). These results further suggest that the gene was *S*-haplotype-specific and located at the *S*-locus (see Text S1 and Fig. S6). The gene encoding SSG3 is hereinafter named *S-LOCUS EARLY FLOWERING 3* (*S-ELF3*). Analysis of nucleotide sequence of 20 alleles of buckwheat *S-ELF3* revealed 42 polymorphic sites among 4,087 nucleotide sites. Nonetheless, no apparent destructive mutations were observed for all 20 *S-ELF3* alleles and all *S* haplotypes were presumed to contain a functional *S-ELF3*. Subsequent RT-PCR analysis revealed that *S-ELF3* is expressed specifically in the pistils and stamens of short-styled flowers, but not in the vegetative tissues, such as the leaves, roots, and stems (Fig. 5). Expression in both the pistils and stamens was confirmed even before flowering.

### The genomic region surrounding *S-ELF3*

Because plant SI is generally controlled by multiple genes located at the *S*-locus, the genomic region surrounding *S-ELF3* may harbor additional genes that are important for heteromorphic SI. Screening of BAC and transformation-competent artificial chromosome (TAC) genomic libraries and subsequent chromosome walking allowed us to construct an *S*-haplotype-specific contig (Fig. S2). High-throughput sequencing analysis of these artificial chromosomes generated about 610 kb of nucleotide sequences in total, although they were divided into 92 fragments due to the difficulty in assembling a contig in the presence of a large number of repetitive elements, including microsatellites. Notably, about one-third of the regions sequenced were occupied by repetitive elements, such as Ty1-copia and Ty3-Gypsy-like retrotransposons. The gene encoding SSG2 was detected within approximately 110∼120 kb of *S-ELF3*. In addition, a few regions were identified by homology search as containing other gene fragments. In particular, fragments homologous to six different genes were detected in two clusters within 10 kb of *S-ELF3*. Most gene fragments identified were nonetheless pseudogenes, since they contained only partial fragments of coding region and/or nonsense mutations in the coding frame. One fragment that lacked any defects was detected in the region 210∼220 kb from *S-ELF3* and encoded a peptide of 109 amino acids that was homologous to hypothetical or predicted conserved proteins of unknown function in various plants (e.g., *Arabidopsis* AT2G26520). However, a homologous gene similar to the one identified above was present and expressed in the plants with long-styled flowers (Fig. S3). Therefore, it is less unlikely to have a primary role in heteromorphic SI.

### *S-ELF3* in other *Fagopyrum* species

**Figure 6:**
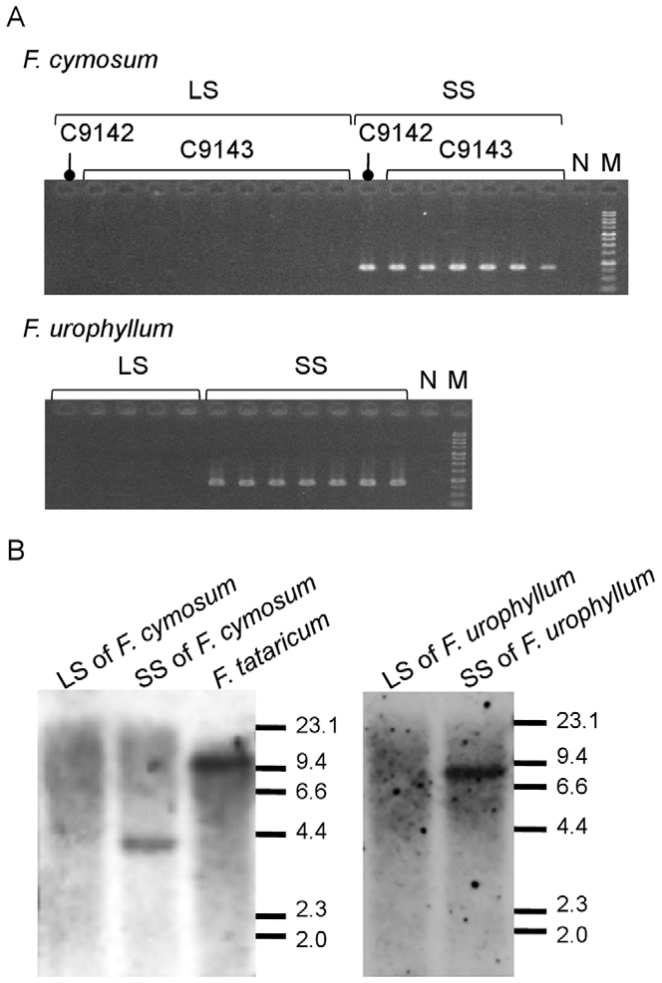
Analysis of *S-ELF3* in *Fagopyurm* species. (A) PCR survey of *S-ELF3* in heteromorphic and self-incompatible species, *F. cymosum* and *F. urophyllum*. LS, plant with long-styled flowers. SS, plant with short-styled flowers. N, negative control. M, molecular marker (1-kb DNA Ladder, GeneDireX) (B) Southern blot analysis of *S-ELF3* in *F. cymosum*, *F. tataricum*, and *F. urophyllum*. Fragment sizes of the λ*Hin*dIII marker shown at the right are in kb. See Table S2 for accession numbers (C9142 and C9143).

**Figure 7:**
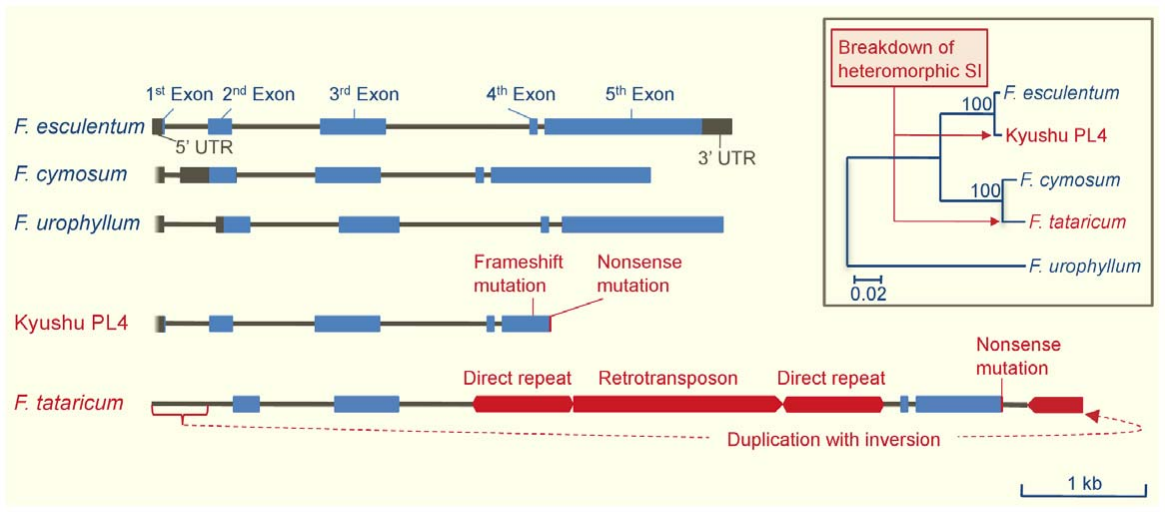
*S-ELF3* in *Fagopyrum* species. The gene structure and phylogeny of *S-ELF3* in five *Fagopyrum* species, including the SC Kyushu PL4 line, which contains the *S^h^* allele of *F. homotropicum*, are shown. Species in blue and red font exhibit heteromorphic SI and homomorphic SC, respectively. Dark brown boxes and lines represent 5′- and 3′-untranslated regions and introns, respectively. Coding regions are colored blue. Red boxes and line indicate large insertions (>400 bp) and nonsense mutation, respectively. The phylogenetic tree in the inset was obtained by the Neighbor-joining method. The *S-ELF3* sequence from *F. urophyllum* was used as an outgroup. The bootstrap numbers (500 replicates) are shown next to the branches. The scale bar corresponds to 0.02 substitution per nucleotide site.

If *S-ELF3* regulates heteromorphic SI and is not merely linked to the *S*-locus, the association between *S-ELF3* and floral morph should be observed even in distantly related *Fagopyrum* species. PCR analysis of 10 long-styled and seven short-styled plants of *F. cymosum* and five long-styled and seven short-styled plants of *F. urophyllum* found that, without exception, all plants possessing *S-ELF3* were of the short-styled morph (Fig. 6A). The results of Southern blot analysis for *F. cymosum* and *F. urophyllum* also show that *S-ELF3* is present only in short-styled plants and not in long-styled plants (Fig. 6B). In addition to the heteromorphic SI species, a few *Fagopyrum* species are known to be homomorphic and SC. Also, SC buckwheat lines, such as Kyukei SC2 and Kyushu PL4, in which the *S^h^* allele of SC and homomorphic *F. homotropicum* species was incorporated into *F. esculentum*, have been produced by embryo rescue of the F_1_ hybrid of *F. homotropicum* and *F. esculentum*, followed by intensive breeding over generations [30]. Analysis of the *S-EFL3* genes in these plants provided further support for the role of *S-EFL3* in heteromorphic SI. In Kyushu PL4, a single nucleotide deletion in the protein-coding region of *S-ELF3* resulted in a frameshift of the 3′ coding region (Fig. 7). *Fagopyrum tataricum*, a homomorphic and SC species, exhibited an inverted duplication of the 5′ region and an insertion of a retrotransposon (Fig. 7). Southern hybridization of *S-ELF3* in these plants indicated that *S-ELF3* is likely a single copy gene (Figs. 3C, 6B) and is incapable of producing functional peptides, since the protein-coding regions were severely damaged, particularly at the conserved C terminus (Fig. S1).

### Genetic Analysis of *SSG2*

Linkage analysis of 1,373 plants failed to identify any recombination events between *SSG2* and the *S*-locus, and PCR analysis of 47 short- and long-styled pairs of buckwheat landraces and modern cultivars showed a complete association between plants with short-styled flowers and the presence of *SSG2* (Fig. S4). These findings are consistent with *SSG2* having a genomic location at the *S*-locus. However, the observation that multiple bands were detected by Southern hybridization analysis of this gene suggests that a gene similar to *SSG2* is present in the genome of long-styled plants. Furthermore, examination of the *SSG2* alleles in 20 short-styled plants identified one (isolated from N8605) that contained a large deletion. The plant that harbored the allele did not have any apparent defects and this cast doubt on the significance of *SSG2* in heteromorphic SI. Furthermore, all pairs of primers tested failed to amplify *SSG2* homologs from other *Fagopyrum* species and Southern hybridization analysis did not detect any homologs of this gene in other species, such as *F. cymosum*. Overall, the current results do not support a role for *SSG2* in heteromorphic SI.

## Discussion

### Persistence of *S-ELF3* among SI plants and its rapid disruption in the SC lineages

In the current study, we observed that, of all three *Fagopyrum* species examined that exhibit heteromorphic SI, only short-styled plants possess *S-ELF3* and the current evidences collectively suggested that *S-ELF3* is located at the *S*-locus. Nucleotide sequence analysis of the protein-coding region of *S-ELF3* of the three *Fagopyrum* species showed that the number of synonymous nucleotide differences per synonymous site is 0.27∼0.29 between the two anciently diverged groups (i.e., the urophyllum group and the cymosum/esculentum group) (Fig. 7). Using 1.0×10^−8^, the synonymous nucleotide substitution rate estimated from the *ELF3* genes of two *Arabidopsis* species, as the nucleotide substitution rate per year for *S-ELF3*, *S-ELF3* appears to have functioned in the plants with short-styled flowers for over 13.5 MY in the genus *Fagopyrum*. On the other hand, the number of synonymous nucleotide differences per synonymous site between the *S-ELF3* genes of *F. esculentum* and Kyushu PL4 is 0.01 and between *F. cymosum* and *F. tataricum* is 0.04, indicating that *S-ELF3* was disrupted recently in the homomorphic SC lineage (Fig. 7). The long persistence of *S-ELF3* on the *S*-locus in the SI plant of *Fagopyrum* and its rapid disruption in the homomorphic SC lineages strongly indicate that *S-ELF3* has an important role in heteromorphic SI in buckwheat.

### Function of *S-ELF3*

Considering that the genomic region around *S-ELF3* contains few functional protein-coding genes and that the functional involvement of genes other than *S-ELF3* in heteromorphic SI remains highly speculative, *S-ELF3* is the most promising candidate gene for controlling heteromorphic SI in buckwheat. Note that the presence of a functional *S-ELF3* gene only in the *S* haplotype is in agreement with the dominance of the haplotype and the diallelism of heteromorphic SI, which can be attained simply by the absence/presence of functional alleles. Floral organ-specific expression of *S-ELF3*, as indicated by RT-PCR analysis (Fig. 5), suggests that it has an important role for the formation of *S* phenotype. Specifically, expression of *S-ELF3* before flowering raised the possibility that S-ELF3 is involved in the development of pistils and stamens of short-styled flowers. To determine the timing of S-ELF3 expression and the cellular location of functional products, tissue in situ hybridization studies of the early stage of flower development, including the flower primodia, is now demanding.

It is interesting to note that *Ath*ELF3 is a nuclear protein with DNA-binding activity that is involved in various processes, including flower timing, circadian rhythms, and photomorphogenesis [38]–[40]. Therefore, it is conceivable that S-ELF3 acts as a transcription factor that is involved in various aspects of heteromorphic SI. Expression of *S-ELF3* in both pistils and stamens strengthens the possibility that it has pleiotropic effects. Considering that the loss of heteromorphy and self-incompatibility occurred simultaneously in the *Fagopyrum* species examined here, the functional role of *S-ELF3* in SI cannot be ruled out.

Regardless of the function of *S-ELF3*, another factor that accounts for the other features of heteromorphic SI must exist, as the male factor of short-styled flowers seems to be intact in the *S^h^* haplotype [30]. The approach taken here, but using stamen-derived RNA and further characterizing the entire *S*-locus for the *S*-haplotype by extending the coverage of contigs will help to identify this factor. In addition, constructing BAC contigs and decoding the genomic region of the *s* haplotype are required to understand the genetic basis of the development of different flower forms. To fully understand heteromorphic SI, including the secondary and downstream pathways, studies that identify any genes operating under the regulation of *S-ELF3* and proteins binding to S-ELF3 are also required.

### Evolution of mating systems

It is remarkable that no recombination was observed between *S-ELF3* and *SSG2* loci in the linkage analysis of 1,373 individuals and that the evolutionary patterns observed for the 20 alleles of *S-ELF3* and *SSG2*, which are separated by about 100 kb, are congruent to each other (Fig. S5), indicating that they were inherited as a single unit. Considering that the sum of branch lengths was 0.010 for the gene tree of 20 *S-ELF3* alleles and applying a value of 1.0×10^−8^ as the nucleotide substitution rate for *S-ELF3*, we estimated that the two genes have coevolved for over ∼1 MY. In the current study, it was noticed that the region an extensive region surrounding *S-ELF3* analyzed here was enriched in transposable elements and numerous pseudogenes, contained pieces of chloroplast DNA, and was inherited as a single unit. Most of these characteristics were also observed in sex chromosomes, including plant sex chromosomes [41], [42]. Even though sex chromosome differentiation was observed in limited plant species, sorrel (*Rumex acetosa*) and its relatives are well-known for possessing identifiable sex chromosomes [43], [44]. Buckwheat was found to be evolutionarily closer to sorrel than to *Koeniga* and *Persicaria* [45], the two genera of *Polygonaceae* in which heteromorphic SI was observed [46]. It has been often argued that plant dioecy has evolved from hermaphroditism via androdioecy, gynodioecy, monoecy or distyly [47]. In *Polygonaceae*, dioecy and heterostyly were observed in plants of several different evolutionary lineages of *Polygonoideae*. In this regard, it is imperative to study the evolution of the heteromorphic SI system in relation to the evolution of dioecy and/or sex determination [26] and to examine if evolution of the heteromorphic SI system, dioecy, and sex chromosome differentiation share some molecular background. Examination of *S-ELF3* in sorrel and other *Polygonaceae* species may therefore shed light on the evolutionary basis of dioecy and/or sex chromosome differentiation. Although the current analysis is the first step in answering questions that have been posed since the time of Darwin, further characterization of the biosystems will provide deep insight into the diversity of plant reproductive systems.

## Materials and Methods

### Plant materials

*Fagopyrum esculentum* of the BC_1_-F_5_ generation, direct descendants of a sib-mating line used to construct a BAC library [31], was utilized to isolate RNA for high-throughput sequence analysis, subjected to ion-beam mutagenesis, and used for linkage analysis. The *F. esculentum* cultivar Kitawase was a gift from the National Agricultural Research Center for Hokkaido region, Japan. The self-compatible line of *F. esculentum* Kyushu PL4, in which the *S^h^* allele was introduced from *F. homotropicum*, was kindly provided by Dr. Matsui of the National Agricultural Research Center for Kyushu Okinawa Region, Japan. Worldwide landraces of *F. esculentum*, *F. cymosum*, and *F. urophyllum*, as well as of *F. tataricum*, collected from 1983 to 1991 by O.O. (Table S2), were used for the association, Southern hybridization, and nucleotide sequencing analyses.

### A chimeric mutant generated by ion-beam mutagenesis

Seeds were irradiated with accelerated ^20^Ne ions (135 MeV/nucleon) in a dose range of 75 to 100 Gy. The linear energy transfer (LET) of ^20^Ne^10+^ was 63.4 keV/µm. Flower morphology was observed for 1,400 M_1_ plants grown in a closed experimental room and a chimeric plant with both types of flower was detected.

### Transcriptome analysis of stylar genes

cDNA from the short and long styles of *F. esculentum* was separately prepared by the method described in Text S2. Oligonucleotide reads generated by a Illumina GAII sequencer were assembled using the Velvet program [48] and analyzed by an in-house program for *in silico* subtraction to select contigs that lacked a 32mer that was present in cDNA isolated from long styles. RT-PCR analysis was conducted using cDNA isolated from short and long styles (Text S2 and Table S1).

### Linkage and association analyses of *F. esculentum S-ELF3*

Linkage analysis of 1,373 *F. esculentum* plants was conducted by testing for the presence of the *S-ELF3* marker by PCR and observing the floral morph, specifically the style and stamen length, after isolating genomic DNA from leaves. Association analysis of the absence/presence of *S-ELF3* and the floral dimorphic morphology was conducted using 47 pairs of short- and long-styled buckwheat plants collected from around the world (Table S2). The PCR conditions used for linkage and association analyses of *S-ELF3* are described in Text S2.

### PCR amplification of *S-ELF3* from *Fagopyrum* species

The same PCR primer pair used for the linkage and association analyses was used to amplify *S-ELF3* from various *Fagopyrum* species, including one sample of *F. tataricum*, 17 samples of *F. cymosum*, and 12 samples of *F. urophyllum* (Text S2, Table S2).

### Evolutionary and sequence analyses

Phylogenetic analysis of the ELF3 homolog and population genetic analysis of *S-ELF3* were conducted using MEGA5 [49]. A synonymous nucleotide substitution rate of *ELF3* genes was inferred using the evolutionary distance (0.108 per site) estimated by the modified Nei-Gojobori method for the genes of two *Arabidopsis* species (GENBANK GI numbers 30682945 and 297822050) that diverged about 5.4 MYA (estimation from www.timetree.org, [50]). The nuclear localization signal motif was predicted using cNLS mapper [51]. Repeat masker [52] (http://www.repeatmasker.org) was used to identify repetitive elements. BLAST programs [53] were used for the homology search.

In the phylogenetic analysis of the conserved region (64 amino acids) of ELF3-related amino acid sequences, the maximum likelihood tree, which was based on the JTT amino acid substitution model [54] with a discrete gamma distribution model for rate differences among sites (parameter = 1.3381), was obtained using MEGA5 [49].

### Basic molecular genetic analysis

The details of 5′ and 3′ RACE, RT-PCR, Southern hybridization, chromosome walking, and sequencing analyses are described in Text S2. Primers used in this study are listed in Tables S1, S3 and S4.

### Data deposition

The DNA sequences have been deposited in the DDBJ/EMBL/GenBank DNA databases under accession numbers AB641416-AB641418 (*S-ELF3*), AB641421-AB641423 (*S-ELF3*), AB642167 (*ELF3*), and AB668583-AB668598 (*SSG2*). The results of high throughput sequence analysis of stylar RNA and pyrosequencing of artificial chromosomes are deposited in the DDBJ Sequence Read Archive (DRA) database under accession number DRA000431.

## Supporting Information

Figure S1 **Buckwheat ELF3 homologs and their phylogenetic relationships.** (A) The deduced amino acid sequences of buckwheat S-ELF3 (SSG3). The conserved peptide motif is shown in bold, the residues that are polymorphic among the 20 alleles examined are shown in red italics, and the predicted monopartite nuclear localization signal, as determined by cNLS Mapper, is underlined. (B) The deduced amino acid sequence of buckwheat *ELF3*. The conserved peptide motif is shown in bold. (C) Phylogenetic tree of ELF3 homologs. The tree is drawn to scale, with branch lengths measured in the number of substitutions per site. The percentage of replicate trees in which the associated proteins clustered together in the bootstrap test (500 replicates) is shown next to the branches. GENBANK GI numbers of amino acid sequences are indicated in parentheses. The homologous sequences from spikemoss *Selaginella moellendorffii* were used as the outgroup.
(TIF)

Figure S2 **A contig map of artificial chromosomes around** ***S-ELF3*** **in buckwheat.** The contig is of the *S* haplotype and contains several gene fragments, mostly pseudogenes(*). Detailed maps of the region surrounding three genes are shown below. Arrows indicate the direction of transcription. 1: homolog of Arabidopsis AT2G26520, 2: SSG2, 3: homolog of hypothetical protein RCOM_0938660*, 4: vacuolar H^+^-pyrophosphatase*, 5: intron of chloroplast trnA-UGC*, 6: cysteine desulfurase*, 7: integral membrane transporter family protein*, 8: S-ELF3, 9: homolog of Arabidopsis AT3G55760*, 10: flagellin-sensitive 2*, 11: embryo-defective 2734*.
(TIF)

Figure S3 **RT-PCR analysis of the gene that is homologous to** ***Arabidopsis*** **AT2G26520.** The actin gene was used as a positive control. LS, plant with long-styled flowers. SS, plant with short-styled flowers.
(TIF)

Figure S4 **PCR survey of** ***SSG2*** **in 47 buckwheat landraces and modern cultivars.** The numbering corresponds to that shown in Table S2. L, long-styled plant. S, short-styled plant. N, negative control. M, XL DNA Ladder 100 bp (APRO).
(TIF)

Figure S5 **Neighbor-Joining trees of 20 buckwheat alleles of (A)** ***S-ELF3*** **and (B)** ***SSG2*** **.** The trees were obtained using MEGA5 and drawn to scale, with branch lengths indicated below. The *p*-distance estimated from 4,087 and 755 nucleotide sites was used for *S-ELF3* and *SSG2*, respectively, and the complete deletion option was applied. The sum of branch lengths was 0.010 for the *S-ELF3* gene tree.
(TIF)

Figure S6 **Breakdown of association between the presence of** ***S-ELF3*** **and floral phenotype by recombination.** Contiguous and dotted lines indicate the frequency of individuals with a positive marker at a locus neighboring *S*-locus in short (*S_x_*) and long-styled (*L_x_*) plants, respectively, under the assumption that the initial population (0^th^ generation) contains only two types of individuals, i.e., long-styled plants, which are homozygotes of the *S-ELF3* ^−^ – *s* haplotype and short-styled plants, which are heterozygotes of the *S-ELF3* ^+^ – *S* and *S-ELF3* ^−^ – *s* haplotypes, with equal frequency.
(TIF)

Table S1 **Contigs obtained by** ***in silico*** **subtraction and pairs of primers used for RT-PCR.**
(DOC)

Table S2 **(A) Heteromorphic and self-incompatible plants used in the study. (B) Homomorphic and self-compatible plants used in the study.**
(DOC)

Table S3 **Primers used for PCR and sequence (seq) analyses.**
(DOC)

Table S4 **Primers used for chromosome walking.**
(DOC)

Text S1 **Supporting information on Results.**
(DOC)

Text S2 **Supporting information on Methods.**
(DOC)
