# Supplementary figures and images for: S-LOCUS EARLY FLOWERING 3 Is Exclusively Present in the Genomes of Short-Styled Buckwheat Plants that Exhibit Heteromorphic Self-Incompatibility
Source: PLoS One. 2012 Feb 1;7(2):e31264. doi: 10.1371/journal.pone.0031264 (PMC3270035; doi:10.1371/journal.pone.0031264)

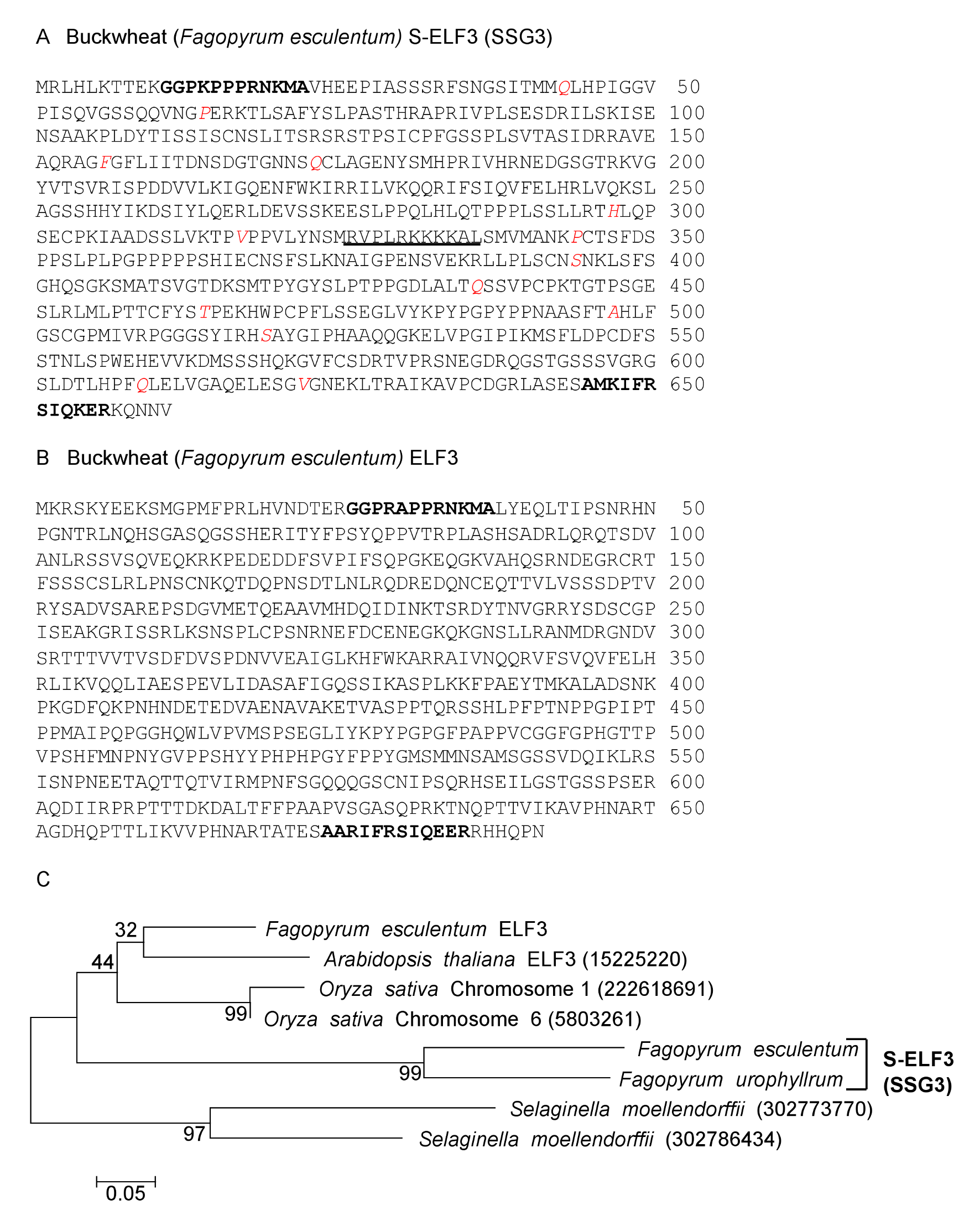

Supplement: Figure S1 — Buckwheat ELF3 homologs and their phylogenetic relationships. (A) The deduced amino acid sequences of buckwheat S-ELF3 (SSG3). The conserved peptide motif is shown in bold, the residues that are polymorphic among the 20 alleles examined are shown in red italics, and the predicted monopartite nuclear localization signal, as determined by cNLS Mapper, is underlined. (B) The deduced amino acid sequence of buckwheat ELF3. The conserved peptide motif is shown in bold. (C) Phylogenetic tree of ELF3 homologs. The tree is drawn to scale, with branch lengths measured in the number of substitutions per site. The percentage of replicate trees in which the associated proteins clustered together in the bootstrap test (500 replicates) is shown next to the branches. GENBANK GI numbers of amino acid sequences are indicated in parentheses. The homologous sequences from spikemoss Selaginella moellendorffii were used as the outgroup. (TIF) [file pone.0031264.s001.tif]

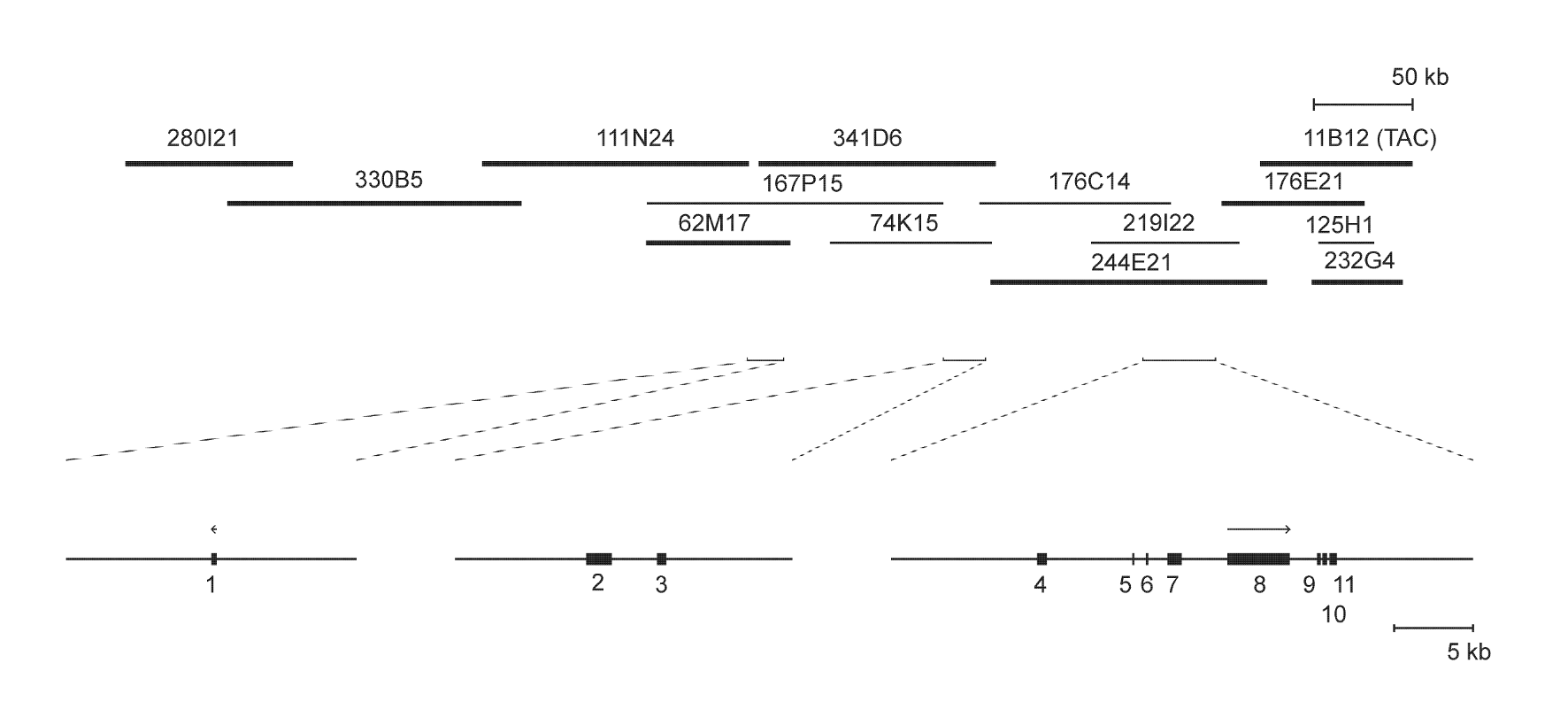

Supplement: Figure S2 — A contig map of artificial chromosomes around S-ELF3 in buckwheat. The contig is of the S haplotype and contains several gene fragments, mostly pseudogenes(*). Detailed maps of the region surrounding three genes are shown below. Arrows indicate the direction of transcription. 1: homolog of Arabidopsis AT2G26520, 2: SSG2, 3: homolog of hypothetical protein RCOM_0938660*, 4: vacuolar H+-pyrophosphatase*, 5: intron of chloroplast trnA-UGC*, 6: cysteine desulfurase*, 7: integral membrane transporter family protein*, 8: S-ELF3, 9: homolog of Arabidopsis AT3G55760*, 10: flagellin-sensitive 2*, 11: embryo-defective 2734*. (TIF) [file pone.0031264.s002.tif]

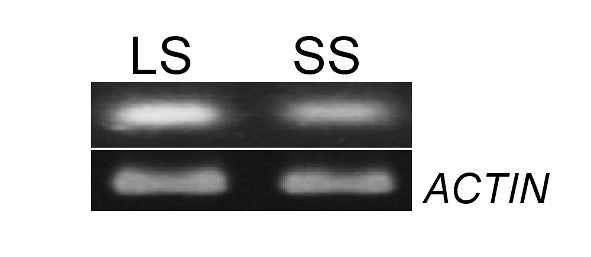

Supplement: Figure S3 — RT-PCR analysis of the gene that is homologous to Arabidopsis AT2G26520. The actin gene was used as a positive control. LS, plant with long-styled flowers. SS, plant with short-styled flowers. (TIF) [file pone.0031264.s003.tif]

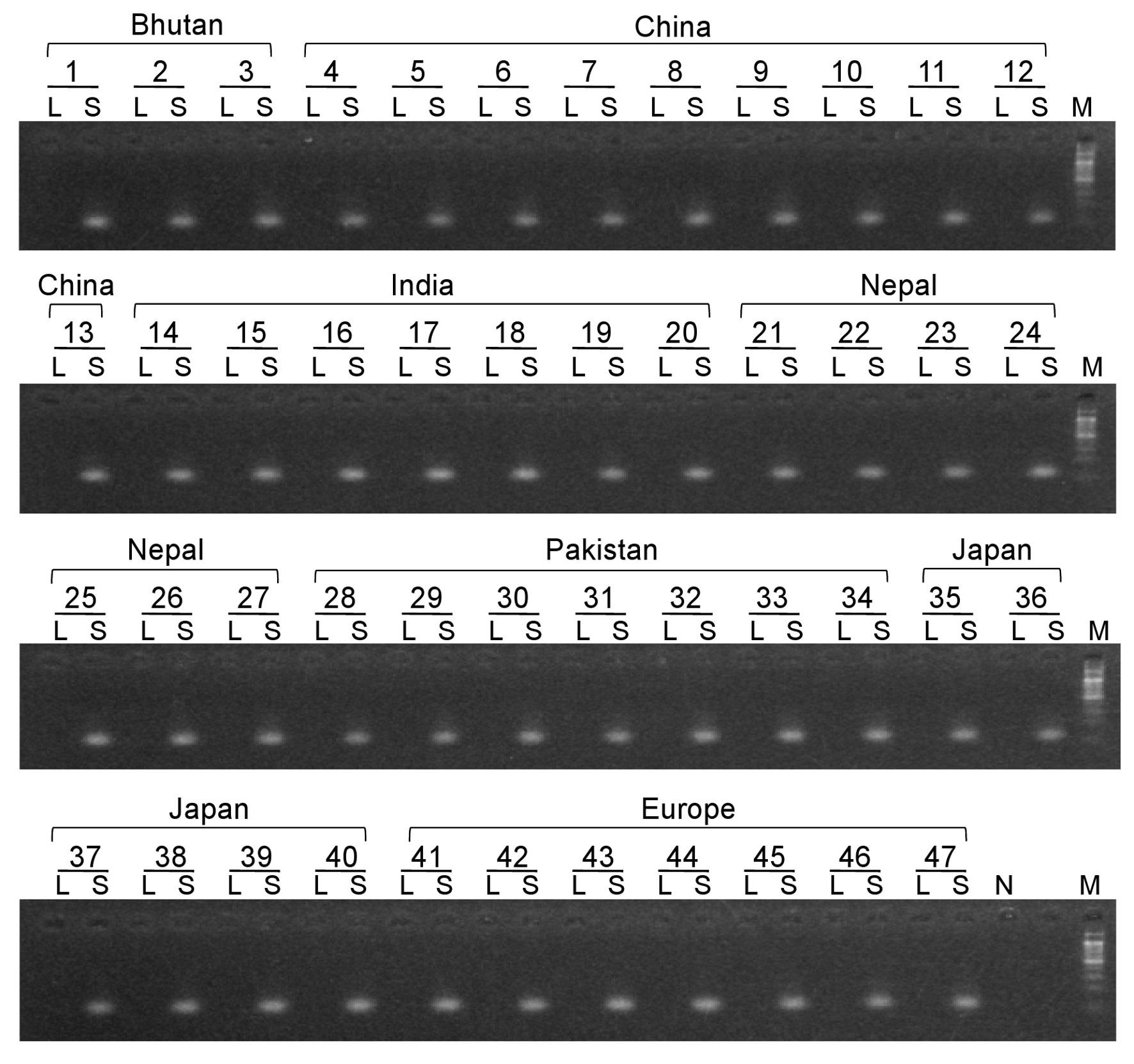

Supplement: Figure S4 — PCR survey of SSG2 in 47 buckwheat landraces and modern cultivars. The numbering corresponds to that shown in Table S2. L, long-styled plant. S, short-styled plant. N, negative control. M, XL DNA Ladder 100 bp (APRO). (TIF) [file pone.0031264.s004.tif]

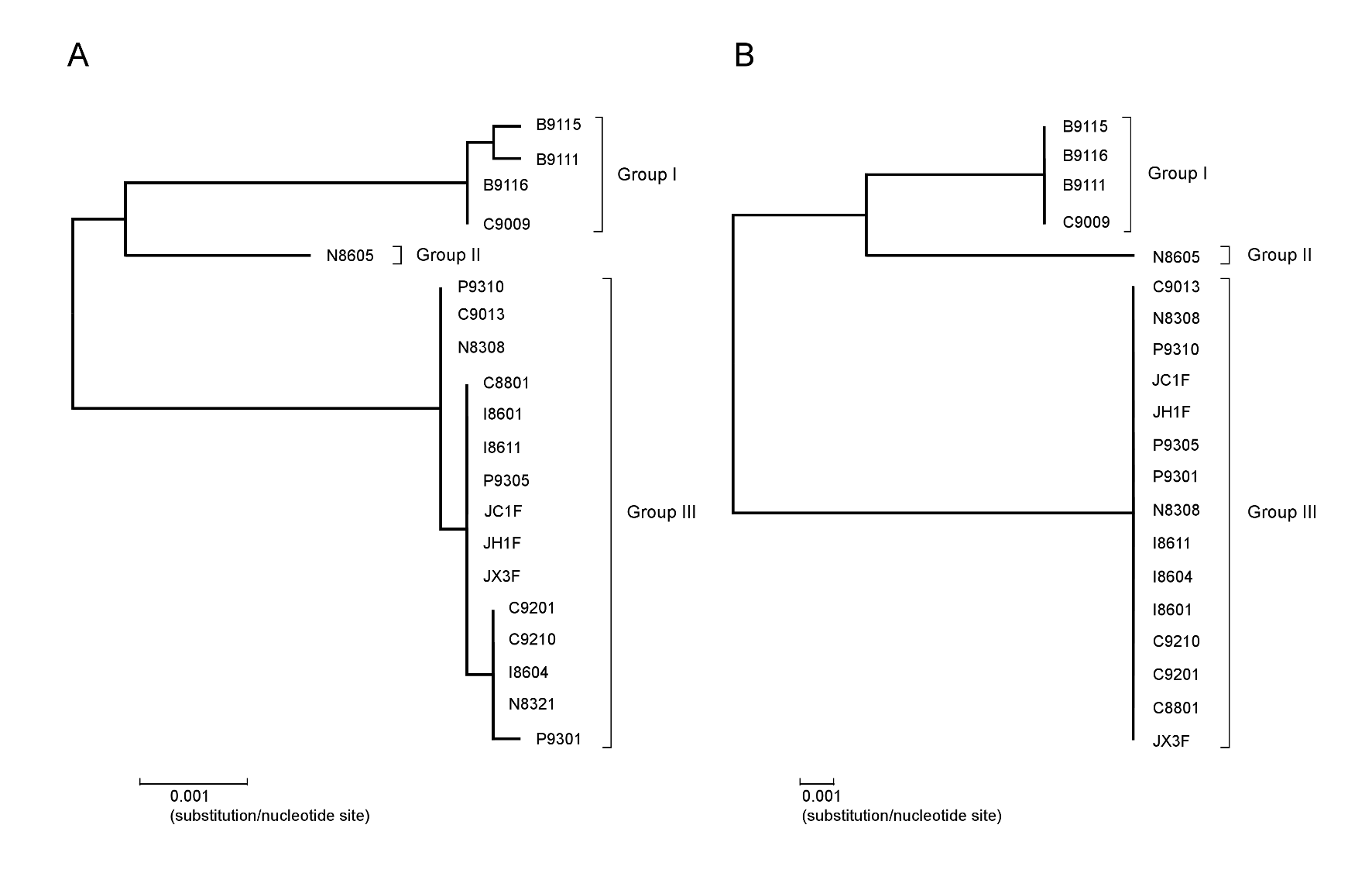

Supplement: Figure S5 — Neighbor-Joining trees of 20 buckwheat alleles of (A) S-ELF3 and (B) SSG2 . The trees were obtained using MEGA5 and drawn to scale, with branch lengths indicated below. The p-distance estimated from 4,087 and 755 nucleotide sites was used for S-ELF3 and SSG2, respectively, and the complete deletion option was applied. The sum of branch lengths was 0.010 for the S-ELF3 gene tree. (TIF) [file pone.0031264.s005.tif]

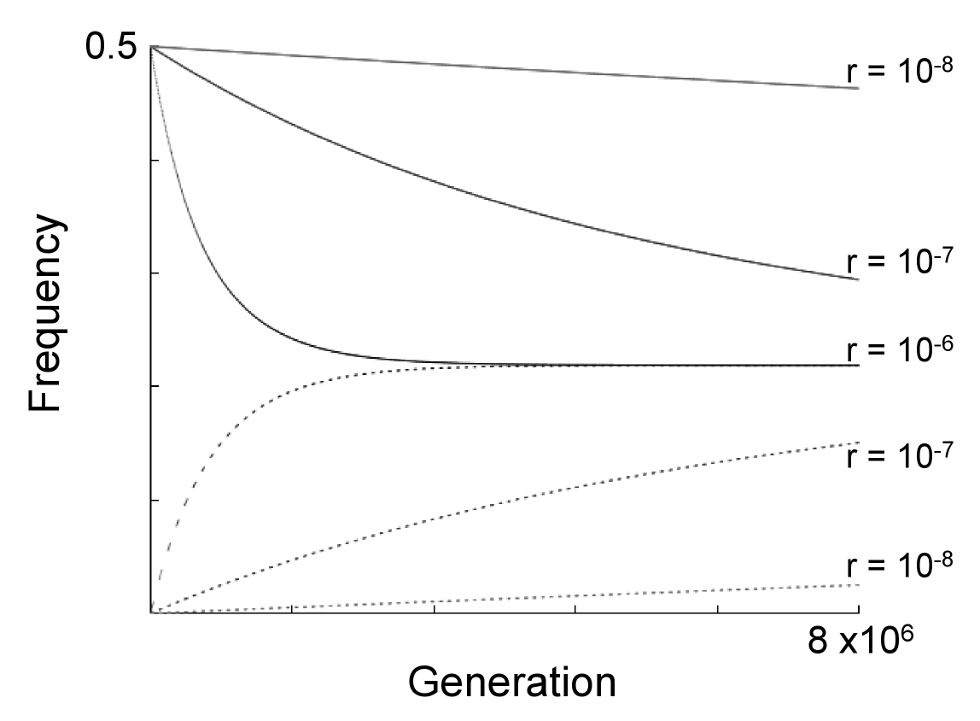

Supplement: Figure S6 — Breakdown of association between the presence of S-ELF3 and floral phenotype by recombination. Contiguous and dotted lines indicate the frequency of individuals with a positive marker at a locus neighboring S-locus in short (Sx) and long-styled (Lx) plants, respectively, under the assumption that the initial population (0th generation) contains only two types of individuals, i.e., long-styled plants, which are homozygotes of the S-ELF3 − – s haplotype and short-styled plants, which are heterozygotes of the S-ELF3 + – S and S-ELF3 − – s haplotypes, with equal frequency. (TIF) [file pone.0031264.s006.tif]
