# Supplementary material for: S-LOCUS EARLY FLOWERING 3 Is Exclusively Present in the Genomes of Short-Styled Buckwheat Plants that Exhibit Heteromorphic Self-Incompatibility
Source: PLoS One. 2012 Feb 1;7(2):e31264. doi: 10.1371/journal.pone.0031264 (PMC3270035; doi:10.1371/journal.pone.0031264)
Supplement: Table S1 — Contigs obtained by in silico subtraction and pairs of primers used for RT-PCR. (DOC) [file pone.0031264.s007.doc]

Table S1 Contigs obtained by *in silico* subtraction and pairs of primers used for RT-PCR.

SSG# Length (bp)* Nucleotide sequences of a pair of Homologous genes identified by BLASTX

primers used for RT-PCR (GENBANK GI number)

1. 132 CGACGCCGAGGGGGATTACGCTAGCGA

GGGTTGCAACGAAGACGAAGGGGA

2. 101 TGGGAAGTGAAAGATGGGTGGAGG

TCTTTCACGTGCCACCTGTCAGCC

3. *SSG1* 140 GAACATCATGTCCACAAGCATGCA IAA-amino acid hydrolase (242073728, 226508210,

TGAAGATTGTATGGGGAGATGCAT 115459478, …)

4. 61 TTAAGATGGCAAAAACACTGTATG

GCTCTTAGTAGATCTGGTTAAGAG

5. *SSG2* 116+94 GTTTTACTGAAAAGTGAGGCTAAAATG

CGACGAATTTGGGATGTAGAGTTTGAA

6. 109 CTCGGCCCGGCTTGTGGAGGCTTT ZINC INDUCED FACILITATOR 1 (255635170, 22326785,

CCCACAAACTGAACGACATTTAGG 297807397, …)

7. *SSG3* 123 TGGAGCTCAAGAGCTGGAGAGTGG EARLY FLOWERING 3 (225442371, 328684593,

(*S-ELF3*) TTCTGTATAGAACGGAATATTTTC 297822051,…)

8. 61 ATTGGGACTAAAGGTTGCATTGTC 50S ribosomal protein L4 (294463048, 297836242,

ATCAAAAACCACAAGCTTCCCTTC 21553676,…)

9. 61 TTATAGAACTATAGGGGGTTTATA

GAGAACACCGTTCGACCCCTTTTT

10. 101 GTCCTTCGAATTCTGCTGGGTA

AGAGAGAAATGGGAGAAGGTCA

11. 61 TTCAACAGCCTGATCAACAAGCTC

TTTGAAAATTGATATTGTTGAGCA

12. 92 GTGAACTTGTGGCTTGTAGGAGGA

GCAAGTGTTAAAGAATTTTTTTGA

13. 145+74+71 GCATGATTCAATAATTAGGGTTCT Predicted/hypothetical protein (224117600, 255544706,

GGTGCCCAAGGCGGTTCCGGGCAT 224114770)

14. 61 TCCTGATGCCACAACCAACGGAGG Ubiquitin-activating enzyme (320166153, 302775296,

GCTCATTCCAGGAGAAACTGCATG 255641903, 255540239, …)

15. *SSG4* 97 CAGGGTTCAACAGAGGCGGCAACA

CCGCTTTCTTGTCCCCTTGAAACG

*: If ambiguous residues of undetermined size were present in the middle of an assembled contig, only the resolved portion of the nucleotide sequences is counted and each region is separated by a plus sign.
