# Supplementary material for: S-LOCUS EARLY FLOWERING 3 Is Exclusively Present in the Genomes of Short-Styled Buckwheat Plants that Exhibit Heteromorphic Self-Incompatibility
Source: PLoS One. 2012 Feb 1;7(2):e31264. doi: 10.1371/journal.pone.0031264 (PMC3270035; doi:10.1371/journal.pone.0031264)
Supplement: Table S2 — (A) Heteromorphic and self-incompatible plants used in the study. (B) Homomorphic and self-compatible plants used in the study. (DOC) [file pone.0031264.s008.doc]

Table S2 (A) Heteromorphic and self-incompatible plants used in the study.

Species Country Number of plants

population number Short-styled plants Long-styled plants

*Fagopyrum esculentum*

1. B9111 Bhutan 1# 1

2. B9115 Bhutan 1# 1

3. B9116 Bhutan 1# 1

4. C8801 China 1# 1

5. C8803 China 1 1

6. C8908 China 1 1

7. C9002 China 1 1

8. C9009 China 1# 1

9. C9013 China 1# 1

10. C9014 China 1 1

11. C9201 China 1# 1

12. C9203 China 1 1

13. C9210 China 1# 1

14. I8601 India 1# 1

15. I8603 India 1 1

16. I8604 India 1# 1

17. I8605 India 1 1

18. I8606 India 1 1

19. I8609 India 1 1

20. I8611 India 1# 1

21. N8308 Nepal 1# 1

22. N8321 Nepal 1# 1

23. N8322 Nepal 1 1

24. N8323 Nepal 1 1

25. N8326 Nepal 1 1

26. N8605 Nepal 1# 1

27. N9109 Nepal 1 1

28. P9301 Pakistan 1# 1

29. P9303 Pakistan 1 1

30. P9304 Pakistan 1 1

31. P9305 Pakistan 1# 1

32. P9308 Pakistan 1 1

33. P9309 Pakistan 1 1

34. P9310 Pakistan 1# 1

35. Kitawase*,+ Japan 1 1

36. Shinano* Japan 1 1

37. JC1F Japan 1# 1

38. JH1F Japan 1# 1

39. JT1F Japan 1 1

40. JX3F Japan 1# 1

41. E1 France 1 1

42. E2 Poland 1 1

43. E3 Russia 1 1

44. E4 Russia 1 1

45. E5 Slovenia 1 1

46. E6 Slovenia 1 1

47. E7 Bosnia and 1 1

Herzegovina

*F. cymosum*

C9142+ China 1# 1

C9143 China 6 9

Table S2 (A) continued

Species Country Number of plants

population number Short-styled plants Long-styled plants

*F. urophyllum*

C8842+ China 7# 5

*: modern cultivar, +:used for Southern hybridization, #:used for nucleotide sequence analysis

Table S2 (B) Homomorphic and self-compatible plants used in the study.

Species Country Number of plants

population number

Hybrids (*Fagopyrum esculentum* and *F. homotropicum*)

Kyushu PL4*,+ Japan 1#

*F. tataricum*

C8816+ China 1#

*: modern cultivar, +:used for Southern hybridization, #:used for nucleotide sequence analysis
