# Supplementary material for: S-LOCUS EARLY FLOWERING 3 Is Exclusively Present in the Genomes of Short-Styled Buckwheat Plants that Exhibit Heteromorphic Self-Incompatibility
Source: PLoS One. 2012 Feb 1;7(2):e31264. doi: 10.1371/journal.pone.0031264 (PMC3270035; doi:10.1371/journal.pone.0031264)
Supplement: Table S3 — Primers used for PCR and sequence (seq) analyses. (DOC) [file pone.0031264.s009.doc]

Table S3 Primers used for PCR and sequence (seq) analyses.

Species Nucleotide sequence Utilization Gene to be analyzed

Primer

*Fagopyrum esculentum*

#13_esc_amp/seq_FW1 GGAGAAAGACGCGTTGAGAGTT PCR/seq *S-ELF3*

#13_esc_amp/seq_RV4600 ACGAAATTCGAGATTTGATTTTAATAG PCR/seq *S-ELF3*

#13_esc_seq_FW660 GTGCTTCCTAGCATATGGATT seq *S-ELF3*

#13_esc_seq_FW1220 AGAATAATTTCATCCGCTTCA seq *S-ELF3*

#13_esc_seq_FW1830 TGGAAAATAAGGCGAATCCT seq *S-ELF3*

#13_esc_seq_FW2020 CACGAGAATACTTAGGGGTGT seq *S-ELF3*

#13_esc_seq_FW3020 CAAAGGATCTTCTCGATTCA seq *S-ELF3*

#13_esc_seq_RV2320 TCCAGTATTCTTAGGGCTGA seq *S-ELF3*

#13_esc_seq_RV2710 TCATATCAATGAAACACTTTACAAC seq *S-ELF3*

#13_esc_seq_RV3320 CAGCGAACTATCAGCTGCAATC seq *S-ELF3*

#13_esc_seq_RV3970 TGTTGTGCAGCATGTGGAA seq *S-ELF3*

#8_esc_amp/seq_FW1 ATGTCTCCCACAAGCAAGAAATCC PCR/seq *SSG2*

#8_ esc_amp/seq_FW115 TACTGGTCTTCCCATATCATGGTTC PCR/seq *SSG2*

#8_ esc_amp/seq_RV1 ATCCCTCACCATTTCAAACTCTACA PCR/seq *SSG2*

Fes_cDNA8_F1(531) CCGGTGCTCATYTGATTCTT seq *SSG2*

Fes_cDNA8_R1(532) TTCTCGGCTTCAAAACCCTA seq *SSG2*

FesELF3_F1(525) GTTGAGGTTGCCTTTTCTCG PCR/seq *ELF3*

FesELF3_F2(526) TATCAGCAAGGGAACCGTCT seq *ELF3*

FesELF3_F3(527) CCTCCTACTCAGCGTTCCAG seq *ELF3*

FesELF3_R1(528) GGAGAGTCAGTGAAATGATCTACATA PCR/seq *ELF3*

FesELF3_R2(529) GCTGGAACGCTGAGTAGGAG seq *ELF3*

FesELF3_R3(530) AGCTGAACGTCCTGCATCTT seq *ELF3*

*F. tataricum*

#13_tat_A_amp/seq_FW7 GGGACAAAGTAAAAGTGAGATCGAG PCR/seq *S-ELF3*

#13_tat_A_amp/seq_RV2643 GAACATGCAGAAGCGAATGTAATAA PCR/seq *S-ELF3*

#13_tat_B_amp/seq_FW2210 TTCTCCAATGCTATGGTCTTCTGTT PCR/seq *S-ELF3*

#13_tat_B_amp/seq_RV6210 CTCGATAAAATGGTAGGATGAACCA PCR/seq *S-ELF3*

#13_tat_C_amp/seq_FW5940 TGAAATACCTTTCTACCTATTTGATCG PCR/seq *S-ELF3*

#13_tat_C_amp/seq_RV8000 GGGCAAATATGAAAATTGTGTGAAG PCR/seq *S-ELF3*

#13_tat_seq_FW580 TTCTGAAAGTGGGTTTTAGGT seq *S-ELF3*

#13_tat_seq_FW6480 GCACTTCTTTCAGTTCACCA seq *S-ELF3*

Table S3 continued.

Species Nucleotide sequence Utilization Gene to be analyzed

primer

*F. tataricum*

#13_tat_seq_FW7022 CATGCTACTCAACTAGGGAAA seq *S-ELF3*

#13_tat_seq_FW7576 GAAAAATTCGGTCCAGGT seq *S-ELF3*

#13_tat_seq_FW1250 TATTCCAGCTTGGACCATAA seq *S-ELF3*

#13_tat_seq_FW1810 CAACGAGCTATCTTGGGTCT seq *S-ELF3*

#13_tat_seq_FW2580 AAAGCGGATTAAAAACCATC seq *S-ELF3*

#13_tat_seq_FW3433 CCGTGACAAGTGGTATCAG seq *S-ELF3*

#13_tat_seq_FW4030 ATTATGGTGGCGTAGAAGGA seq *S-ELF3*

#13_tat_seq_FW4595 ATTGTCCGACGAAAAACAAG seq *S-ELF3*

#13_tat_seq_RV3770 TCAAACTTGGCCTTGAGAAC seq *S-ELF3*

#13_tat_seq_RV5920 AATAGCTACAGAAAAAGATACTAGTG seq *S-ELF3*

*F. cymosum*

#13_esc_amp/seq_FW1 GGAGAAAGACGCGTTGAGAGTT PCR/seq *S-ELF3*

#13_cym_amp/seq_RV3880 CATATTACTGTGATCCATTCCGCTAC PCR/seq *S-ELF3*

#13_cym_seq_FW_1350 CCAGATCCAGTTCTACACCA seq *S-ELF3*

#13_cym_seq_FW_1822 GCATGAAAATCGTCGATAAA seq *S-ELF3*

#13_cym_seq_FW530 CTCGACAGATGCTCTTCATT seq *S-ELF3*

#13_cym_seq_FW830 AAACAACAACAATCTGGCTAC seq *S-ELF3*

#13_cym_seq_FW2230 AAGCGGATTAAAAACCATCA seq *S-ELF3*

#13_cym_seq_RV3190 AGTCTCCAGAAGGTGTTCCA seq *S-ELF3*

#13_cym_seq_RV3370 ACCTGGAGGGACATTCAT seq *S-ELF3*

*F. urophyllum*

#13_uro_amp/seq_FW60 GTGATCTGTTGAGAGTTCTTCGTCA PCR/seq *S-ELF3*

#13_uro_amp/seq_RV4970 TACCGTTCTTTTGCCCCTATTTAAG PCR/seq *S-ELF3*

#13_uro_seqFW_2336 GTGAGGCTACGCATGTATGA seq *S-ELF3*

#13_uro_seq_FW640 GACTACATGTGAAAGACACCGAGAAA seq *S-ELF3*

#13_uro_seq_FW1330 TCAAGACACACGGCCAACAA seq *S-ELF3*

#13_uro_seq_FW1820 ACCAATCTTGCCGTTAGG seq *S-ELF3*

#13_uro_seq_RV3160 ACGATGAGAAATTGAGAACTG seq *S-ELF3*

#13_uro_seq_RV3840 TGCTGCTAAGAGGAGGAAGT seq  *S-ELF3*

#13_uro_seq_RV4360 CTCATGTCTTTAATCGCTGCT seq *S-ELF3*
