# Supplementary material for: S-LOCUS EARLY FLOWERING 3 Is Exclusively Present in the Genomes of Short-Styled Buckwheat Plants that Exhibit Heteromorphic Self-Incompatibility
Source: PLoS One. 2012 Feb 1;7(2):e31264. doi: 10.1371/journal.pone.0031264 (PMC3270035; doi:10.1371/journal.pone.0031264)
Supplement: Table S4 — Primers used for chromosome walking. (DOC) [file pone.0031264.s010.doc]

Table S4 Primers used for chromosome walking.

Primer name Nucleotide sequence

330B5_RVN_FW34 TATGTTGAGGATACAATGGTCGAGA

330B5_RVN_RV302 GAGGAAATAACCATCTCCATCCATA

111N24_RVN_FW59 ACACCAATAGTAATGCCTCACGTTC

111N24_RVN_RV306 ATTCTGTGGCGGATCACCTTAGTA

62M17_RVN_FW21 ACCACAGAGAAAATACAAGATGTGG

62M17_RVN_RV332 TTCTGCAGTTGAGAGTAATCCAGTG

341D6_RVN_FW3 GCCCGTATTTCTATCACCAACTACA

341D6_RVN_RV363 TCATACGTTCTTCATCTCCTCCTTT

74K15_RVN_FW55 AACAAGCCAAACAGAAGATACGAAC

74K15_RVN_RV461 CCTACCCGGTGATTCTTACTCTTAG

244E21_RVN_FW91 ATACATGTCATCACGAGGAACGAT

244E21_RVN_RV361 TTCTTTATGACTTGTGCTTGTGCTT

176E21_M4_FW33 AAGTGTACAGCTGGGCATCTTCAT

176E21_M4_RV247 TGCATGAATGGAAAGGAAAACTATC

232G4_M4_FW6 CAGCTCCTATCATTTCGAGTCCTCT

232G4_M4_RV307 TCAACACAAACAAGCAAACAATACA
