# Supplementary material for: S-LOCUS EARLY FLOWERING 3 Is Exclusively Present in the Genomes of Short-Styled Buckwheat Plants that Exhibit Heteromorphic Self-Incompatibility
Source: PLoS One. 2012 Feb 1;7(2):e31264. doi: 10.1371/journal.pone.0031264 (PMC3270035; doi:10.1371/journal.pone.0031264)
Supplement: Text S1 — Supporting information on Results. (DOC) [file pone.0031264.s011.doc]

**Supporting information**

**Genetic linkage between *S-ELF3* and the *S-locus.***

In the current study, we observed that, of all three *Fagopyrum* species examined that exhibit heteromorphic SI, only short-styled plants possess *S-ELF3*. Assuming that two separate loci encode *S-ELF3* and the *S*-locus, that random mating occurs between plants with different flower morphs, and that the frequency of short-styled and long-styled plants in a population is equal, the frequency of individuals with *S-ELF3* in short- (S) and long-styled (L) plants at equilibrium is expected to be

$S=\frac{1}{2}-\frac{8}{3}(\frac{1}{4}-P_{eq})O_{eq}$ and $L=\frac{1}{2}-\frac{8}{9}O_{eq}^{2}$,

respectively, where *P_eq_* and *O_eq_* are the haplotype frequency of the *S-ELF3*^+^ – *S* and *S-ELF3*^-^ – *s* haplotypes, respectively (note that *S-ELF3*^+^ – *S* means that the haplotype has both the *S-ELF3* and *S* allele). Noting that the haplotype frequency of the *S-ELF3*^-^ – *S* and *S-ELF3*^+^ – *s* haplotypes is 0.25 – *P_eq_* and 0.75 – *O_eq_*, respectively, and that two-thirds of the *S-ELF3*^-^ – *s* and *S-ELF3*^+^ – *s* haplotypes are present in long-styled plants at equilibrium, the expected frequency of individuals without *S-ELF3* in long-styled plants is given by

$(\frac{2}{3}O_{eq}/\frac{1}{2})^{2}=\frac{8}{9}O_{eq}^{2}(=\frac{1}{2}-L)$.

As shown in the Results section, *S* = 0.5 and *L* = 0 in all three *Fagopyrum* species with heteromorphic SI; therefore, *P_eq_* = 0.25 and *O_eq_* = 0.75. It is then concluded that only *S* haplotypes, but not *s* haplotypes, contain functional *S-ELF3* alleles. It is furthermore reasonable to presume that the ancestral species of the genus exhibiting heteromorphic SI contains only two types of individuals, i.e., long-styled plants, which are homozygotes of the *S-ELF3*^-^ – *s* haplotype and short-styled plants, which are heterozygotes of the *S-ELF3*^+^ – *S* and *S-ELF3*^-^ – *s* haplotypes.

In general, over long periods of time, genetic recombination would break the association between the presence of *S-ELF3* and floral morphology, if *S-ELF3* and the gene controlling floral morphology are distinct loci. Theoretically, assuming that the recombination rate between the two loci is *r* per generation ($0\leq r\leq0.5$), the expected frequency of short-styled (*S_x_*) and long-styled (*L_x_*) individuals with *S-ELF3* after *x* generations is given by

$S_{x}=\frac{1}{2}-\frac{1}{4}(1-4p)(1+4p+4q)$ and $L_{x}=\frac{1}{2}-\frac{1}{4}(1-4q)(1+4p+4q)$,

respectively, where

$p=\frac{1}{16}+\frac{3}{32}(k_{1}^{x}+k_{2}^{x})+\frac{(9-10r)(k_{1}^{x}-k_{2}^{x})}{64(k_{1}-k_{2})}$,

$q=\frac{1}{16}-\frac{1}{32}(k_{1}^{x}+k_{2}^{x})-\frac{(3-14r)(k_{1}^{x}-k_{2}^{x})}{64(k_{1}-k_{2})}$,

and

$k_{1}=(\frac{1}{4}-\frac{r}{2})+\sqrt{(\frac{1}{4}-\frac{r}{2})(\frac{9}{4}-\frac{r}{2})}$,

$k_{2}=(\frac{1}{4}-\frac{r}{2})-\sqrt{(\frac{1}{4}-\frac{r}{2})(\frac{9}{4}-\frac{r}{2})}$ .

As illustrated in Figure S6, even a low rate of recombination is expected to break the association between the absence/presence of *S-ELF3* and the floral dimorphic morphology over the history of the genus *Fagopyrum*. For example, if the two genes were separated by 10 kb and located at a region where recombination is highly suppressed (0.018 cM/Mb), such as that in the *Arabidopsis* *S*-locus (1), *r* would have a value of 1.8 x 10^-6^, and the association found here would not be observed. Taking this into consideration, we conclude that *S-ELF3* is located at the *S*-locus.

**References**

1. Kamau E, Charlesworth B, Charlesworth D (2007) Linkage disequilibrium and recombination rate estimates in the self-incompatibility region of *Arabidopsis* *lyrata*. Genetics 176: 2357-2369.
