# Supplementary material for: S-LOCUS EARLY FLOWERING 3 Is Exclusively Present in the Genomes of Short-Styled Buckwheat Plants that Exhibit Heteromorphic Self-Incompatibility
Source: PLoS One. 2012 Feb 1;7(2):e31264. doi: 10.1371/journal.pone.0031264 (PMC3270035; doi:10.1371/journal.pone.0031264)
Supplement: Text S2 — Supporting information on Methods. (DOC) [file pone.0031264.s012.doc]

**Supporting information**

**SI Materials and Methods**

**Transcriptome analysis of stylar RNAs.**

Total RNA was extracted from bulks of 200 styles of 15 plants of either short-styled or long-styled flowers using the method developed by Chang et al. (1). Upon purification of total RNA using the SV Total RNA Isolation Kit (Promega), cDNA was synthesized and sequence libraries were constructed using the mRNA-Seq Sample Preparation Kit (Illumina) following the manufacturer’s protocol. Sequence libraries were analyzed by the Illumina GAII instrument using one lane with a read length of 50nt and one lane with a read length of 51nt for long styles and one lane with a read length of 50nt for short styles. Oligonucleotide reads were assembled by the Velvet program (2) and the assembled contigs were examined by in-house programs if they contained any 32mer motif observed in the oligonucleotide reads for long styles in order to select possible fragments of short style specific genes. In this in silico subtraction, not only the oligonucleotide reads composing the assembled contigs but also their paired-reads were examined if they were observed in oligonucleotide reads for long styles to increase the efficiency of subtraction. Fifteen assembled contigs were selected by this in silico subtraction procedure. The programs used are available from the authors upon request. To verify the short style-specific expression of transcripts, PCR primers were prepared (Table S1) and RT-PCR analysis was conducted using KOD-Plus-Ver.2 DNA Polymerase (Toyobo) and template cDNA isolated from short- and long-styles. The actin gene was amplified as a positive control using the following primers: 5’ primer GGCATCACACTTTCTACAATGAGC and 3’ primer GATATCCACATCACATTTCATGAT. The PCR conditions used were as follows: 94°C for 2 min, followed by 30 cycles of 98°C for 5 s, 55°C for 5 s, and 68°C for 5 s. PCR products were separated on 2% agarose gels and stained with ethidium bromide, and gel images were captured using a UV illuminator.

**PCR analysis of *SSG1*-*SSG4***.

PCR was performed using PrimeStar DNA polymerase (Takara) and 100 ng of genomic DNA isolated separately from the sections of the ion-beam-induced chimeric mutant with short-styled and long-styled flowers. The PCR primers used were as follows: *SSG1*, 5’ primer GAACATCATGTCCACAAGCATGCA and 3’ primer TGAAGATTGTATGGGGAGATGCAT; *SSG2*, 5’ primer GTTTTACTGAAAAGTGAGGCTAAAATG and 3’ primer CGACGAATTTGGGATGTAGAGTTTGAA; *SSG3*, 5’ primer TGGAGCTCAAGAGCTGGAGAGTGG and 3’ primer TTCTGTATAGAACGGAATATTTTC; *SSG4*, 5’ primer CAGGGTTCAACAGAGGCGGCAACA and 3’ primer CCGCTTTCTTGTCCCCTTGAAACG. The PCR conditions used were as follows: 98°C for 3 min, followed by 30 cycles of 98°C for 10 s and 68°C for 1 min. PCR products were separated on 4% NuSieve 3:1 Agarose gels (Takara) and stained with ethidium bromide.

**Southern hybridization analysis.**

Five micrograms of genomic DNA, isolated from the Kitawase cultivar, Kyushu PL4 line, *Fagopyrum cymosum* (C9142), *F. tataricum* (C8816) or *F. urophyllum* (C8842), was digested with the restriction enzyme *Hind*III, fractionated on a 0.8% agarose gel, and blotted onto nylon membrane (Byodyne Plus, PALL). The hybridization probe for *S-ELF3* was prepared using the PCR Dig Probe Synthesis Kit (Roche) with 5’ primer CACCACCTCCATCACATATT, 3’ primer TTTGCCCATTCAGTTATCCT, and template DNA isolated from a short-styled plant of the Kitawase cultivar or *F. urophyllum*. The hybridization probe for *SSG2* was prepared with 5’ primer ATGTCTCCCACAAGCAAGAAATC, 3’ primer AAGGGAAGCCCAATAACCTAGTTC, and template DNA isolated from a short-styled plant of the Kitawase cultivar. Hybridization was conducted using the DIG Easy Hyb (Roche) at 42°C for 16 h. After hybridization, membranes were washed twice with low stringency buffer (0.5X SSC and 0.1% SDS) for 5 min at 25°C and twice with high stringency buffer (0.1X SSC and 0.1% SDS) for 15 min at 65°C. Dig signal was detected using a DIG Wash and Block Buffer Set (Roche), according to the manufacturer’s protocol.

**5’ and 3’ RACE of *S-ELF3*.**

RACE PCR was performed using the SMARTer PCR cDNA Synthesis Kit (Takara). A specific primer, CGGATTGATGCCCAGAGAACGAGAG, and a nested primer, TAGAGGACAGGAGGAACGGGGGTCT, were used for 5’ RACE, and a specific primer, CTGGTTGGAGCTCAAGAGCTGGAGAGT, and a nested primer, ATCTGCAATGAAAATATTCCGTTCT, were used for 3’ RACE. After 5’ and 3’ RACE, full-length cDNA was amplified with 5’ primer GGAGAAAGACGCGTTGAGAGTT and 3’ primer ACGAAATTCGAGATTTGATTTTAATAG, using PrimeStar DNA polymerase (Takara) under the following conditions; 98°C for 3 min, followed by 30 cycles of 98°C for 5 s, 60°C for 5 s, and 72°C for 3 min, and 72°C for 5 min. PCR products were cloned into the TOPO vector using the Zero Blunt TOPO PCR Cloning Kit for Sequencing (Invitrogen). Once inserts of the appropriate size were confirmed, nucleotide sequences were determined using a 3130 DNA sequencer (Applied Biosystems). Using the same PCR primers to amplify full-length cDNA, the genomic region of *S-ELF3* was amplified from short-styled plants of *F. esculentum*. The PCR products were directly sequenced using a 3130 DNA sequencer (Applied Biosystems) with various sequence primers (Table S3).

**RT-PCR analysis of *S-ELF3* expression in various organs of *F. esculentum*.** Total RNA was extracted from the roots, leaves, pistils, stamens, and pollen of the Kitawase cultivar. Pistils and stamens were removed from flowers at the one day before flowering and at the day of flowering. Single strand cDNA synthesized using the SuperScript VILO cDNA Synthesis Kit (Invitrogen) was used as template for PCR. PCR was performed using the following primers: the 5’-primer TGATTTTGCGTATGTATGAGGTTGC and 3’-primer GATTCAGACAAAGGGACAATCCTTGG for *S-ELF3*; and 5’-primer GGCATCACACTTTCTACAATGAGC and 3’-primer GATATCCACATCACATTTCATGAT for *ACTIN*. KOD-Plus-Ver.2 DNA Polymerase (Toyobo) was used and PCR conditions were as described in the section on “Transcriptome analysis of stylar RNAs”.

**RT-PCR of *ELF3*.**

The 5’ primer TTGAGGTTGCCTTTTCTCGT and 3’ primer GGAGAGTCAGTGAAATGATCTACATA were designed based on the results of the transcriptome analysis of stylar cDNAs and were used to amplify buckwheat *ELF3*. PCR were performed with ExTaq (Takara) and cDNA isolated from short-styled flowers as template with the following conditions: 94°C for 2 min, followed by 45 cycles of 94°C for 30 s, 60°C for 10 s, and 72°C for 1 min, and then 72°C for 10 min. RT-PCR products were directly sequenced using a 3130xl DNA sequencer (Applied Biosystems) and various sequence primers (Table S3).

**Linkage and association analysis of *S-ELF3* for *F. esculentum***.

Linkage analysis of *S-ELF3* was conducted by testing for the presence of *S-ELF3* by PCR and examining flower morphology, especially style and stamen length, in 1,373 individuals of a sib-mating line. PCR was performed using PrimeStar DNA polymerase (Takara), template DNA isolated from leaves, 5’ primer ACGGAATATTTTCATTGCAGATTCA, and 3’ primer GGGGTTGGCAATGAGAAACTAAC. The conditions used were as follows: 98°C for 3 min, followed by 30 cycles of 98°C for 5 s, 60°C for 5 s, and 72°C for 30 s, and then 72°C for 5 min. Association analysis of *S-ELF3* was conducted with genomic DNA isolated from 47 pairs of short- and long-styled buckwheat plants collected from all over the world (Table S2).

**Construction of contigs by chromosome walking.**

To investigate the chromosomal region harboring *S-ELF3*, the bacterial artificial chromosome (BAC) library (3), equivalent to a seven- to eight-fold coverage, and a transformation-competent artificial chromosome library, equivalent to a 1.3-fold coverage, were screened. Once positive clones were identified by PCR-based screening, the nucleotide sequences of both ends of insert DNA were determined using a 3130 sequencer (Applied Biosystems) and new pairs of PCR primers were designed for the next walking step. To construct a *S*-haplotype-specific contig, pairs of primers were designed to amplify only from the short-styled plants of the sib-mating line (Table S4). PCR screenings were conducted using KOD plus DNA polymerase (Toyobo) with the following conditions: 94°C for 3 min, 30 cycles of 94°C for 10 s, 60°C for 15 s, and 68°C for 30 s, and then 68°C for 5 min.

**Pyrosequencing of BAC/TAC clones.**

Isolated plasmid DNAs of BAC/TACs were analyzed in bulk using the FLX 454 sequencer (Roche), according to the manufacturer‘s protocol. After excluding nucleotide sequences of vectors and *E. coli* contaminants, nucleotide sequences were assembled using the GS Denovo Assembler v2.3. Once repetitive elements were removed by RepeatMasker (http://www.repeatmasker.org/), homology search by BLAST (4) was conducted to identify gene fragments present in the region. For the detected homolog of AT2G26520, RT-PCR analysis using cDNA isolated from short- and long-styles was conducted separately. PCR was performed using 5’ primer TCCTCTGTGCCGTGGCTGTA and 3’ primer ACCGCCGTTGACAGAGCTTC and the conditions described in the section of Transcriptome analysis of stylar RNAs.

**PCR amplification and DNA sequencing of *S-ELF3* for *F. cymosum*, *F. tataricum*, and *F. urophyllum***.

Based on the nucleotide sequences of *S-ELF3* of *F. esculentum*, various pairs of primers were designed. Products of the appropriate size were obtained from short-styled plants of *F. cymosum*, *F. tataricum*, and *F. urophyllum* using 5’ primer TTGTGTACAAGCCCTACCCTGGA, 3’ primer AGAAGCGAGTCTCCCATCACAAG, and PrimeStar DNA polymerase (Takara) and the following conditions: 98°C for 3 min, followed by 30 cycles of 98°C for 5 s, 58°C for 5 s, and 72°C for 30 s, and then 72°C for 5 min. Under these same conditions, association analysis of *S-ELF3* was conducted for 17 individuals of *F. cymosum* and 12 individuals of *F. urophyllum* (Table S2). The franking regions of *S-ELF3* of *F. cymosum*, *F. tataricum* and *F. urophyllum* were obtained by inverse PCR, as described in Fawcett et al. (5). Full-length *S-ELF3* genes of *F. cymosum* and *F. urophyllum* were generated using a pair of primers that were based on the results of inverse PCR. *S-ELF3* of *F. tataricum* contained long terminal repeats as well as inverted repeats and was amplified in three separate parts individually. To determine the precise gene structure of exon/intron junctions, RT-PCR analysis of *S-ELF3* was conducted using cDNA obtained from the short styles of *F. cymosum* and *F. urophyllum* as template and their products were cloned into the TOPO vector and sequenced.

**5’ and 3’ RACE for *SSG2*.**

The method described for 5’ and 3’ RACE of *S-ELF3* was applied to the analysis of *SSG2*. A specific primer, ACTTGAAGTCGCGCAAGCTTTATGGAAT, and a nested primer, TATCCCTCACCATTTCAAACTC, were used for 5’ RACE, and a specific primer, GACAACCGGGATATTCCATAAAGCTTGC, and a nested primer, GCATTTTAGCCTCACTTTTCAG, were used for 3’ RACE. After 5’ and 3’ RACE, full-length cDNA was amplified with 5’ primer ATGTCTCCCACAAGCAAGAAATCC and 3’ primer TATATTCTAGCCACAAAACCCAGCA. PCR products were cloned into a TOPO vector and sequenced with additional sequencing primers, ACCTTTCTTACTTGCTCTTGG and AACTCTACATCCCAAATTCGTC.

**Sequence analysis of *S-ELF3* and *SSG2*.**

The nucleotide sequences of *S-ELF3* and *SSG2* were obtained for twenty short-styled buckwheat landraces (Table S2) by directly sequencing the corresponding PCR products. For *S-ELF3*, PCR was performed using 5’ primer GGAGAAAGACGCGTTGAGAGTT, 3’ primer ACGAAATTCGAGATTTGATTTTAATAG, and PrimeStar DNA polymerase (Takara) with the following conditions; 98°C for 3 min, followed by 30 cycles of 98°C for 5 s, 60°C for 5 s, and 72°C for 4 min, and then 72°C for 5 min. For *SSG2*, PCR was performed using 5’ primer ATGTCTCCCACAAGCAAGAAATCC, 3’ primer ATCCCTCACCATTTCAAACTCTACA, and PrimeStar DNA polymerase (Takara) with the following conditions; 98°C for 3 min, followed by 30 cycles of 98°C for 5 s, 60°C for 5 s, and 72°C for 2 min, and then 72°C for 5 min. For one plant (C8801) in which the above procedure failed, 5’ primer TACTGGTCTTCCCATATCATGGTTC was used instead. The PCR product obtained was directly sequenced using a 3130 DNA sequencer or a 3130xl DNA sequencer (Applied Biosystems) with various sequence primers (Table S3).

**References**

1. Chang S, Puryear J, Cairney J (1993) A simple and efficient method for isolating RNA from pine trees. Plant Mol Biol Rep 11: 113-116.

2. Zerbino DR, Birney E (2009) Velvet: algorithms for de novo short read assembly using de Bruijn graphs. Genome Res 18: 821-829.

3. Yasui Y, Mori M, Matsumoto D, Ohnishi O, Campbell CG et al. (2008) Construction of a BAC library for buckwheat genome research—an application to positional cloning of agriculturally valuable traits. Genes Genet Syst 83: 393–401.

4. Altschul SF, Gish W, Miller W, Myers EW, Lipman DJ (1990) Basic local alignment search tool. J Mol Biol 215: 403–410.

5. Fawcett JA, Kawahara T, Watanabe H, Yasui Y (2006) A SINE family widely distributed in the plant kingdom and its evolutionary history. Plant Mol Biol 61: 505-514.
